# Dopaminergic Agents in Rheumatoid Arthritis

**DOI:** 10.1007/s11481-019-09850-5

**Published:** 2019-04-23

**Authors:** Silvia Capellino

**Affiliations:** Junior Group Neuroimmunology, Department of Immunology, IfADo - Leibniz Research Centre for Working Environment and Human Factors at TU Dortmund, Ardeystrasse 67, 44139 Dortmund, Germany

**Keywords:** Dopamine, Dopaminergic receptors, Rheumatoid arthritis

## Abstract

Clinical evidences suggest a causal relationship between rheumatoid arthritis (RA) and the dopaminergic system, and several studies described an alteration of the disease in patients treated with dopaminergic agents. Despite these interesting results, potential direct effects of dopamine on RA have not been intensively considered until the last decade. Recent studies confirm a direct effect of dopamine on the systemic immune response as well as on bone remodeling and on joint inflammation, both in humans and in different animal models of arthritis. While more research is necessary to accurately determine the effect of dopamine in RA, these results are encouraging and support a possible use of dopaminergic drugs for the treatment of arthritis in the future. Moreover, they point out that dopaminergic agents use to treat comorbidities, might influence the immune response and the disease progression in RA patients. This review summarizes the current knowledge about the effects of dopaminergic drugs on RA and describes the potential of dopaminergic drugs as future therapeutic strategy in arthritis.

**Figure:**
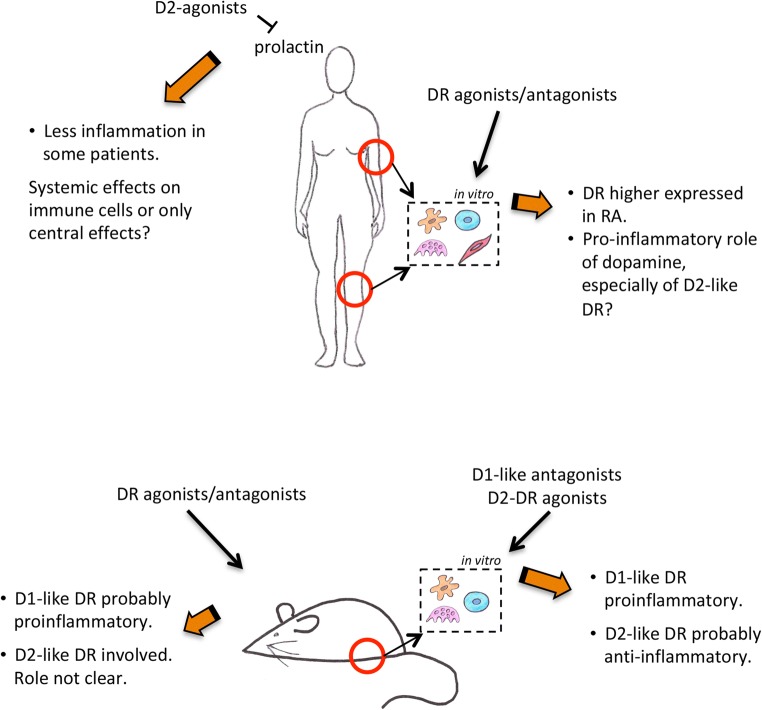
Graphical Abstract

Graphical Abstract

## Introduction

Rheumatoid arthritis (RA) is an autoimmune disease characterized by chronic joint inflammation, articular bone erosion and consequently joint destruction that can lead to complete loss of function (Smolen et al. 2018). Joint inflammation in RA affects multiple sites of the human organism causing widespread pain. The subsequent joint destruction can lead to severe disability affecting all aspects of motor function, from walking to fine movements of the hand (In. Rheumatoid Arthritis: National Clinical Guideline for Management and Treatment in Adults. London 2009). Moreover, RA is not just a disease of the joints but can affect many other organs and cause, for instance, systemic and localized osteoporosis (Dubrovsky et al. 2018), vasculitis and cardiovascular diseases (Romano et al. 2018), and lung fibrosis (Paulin et al. 2017), thus leading to an increased risk of mortality.

Clinical evidences suggest an involvement of the dopaminergic system in RA. For instance, in schizophrenia patients, treated with dopamine receptor (DR) antagonists, the incidence of RA is substantially lower than in the general population (Sellgren et al. 2014; Baldwin 1979). A possible interplay between RA and Parkinson’s disease was also hypothesized, even though the results are controversial (Sung et al. 2016; Bes et al. 2014). In addition, RA patients often develop restless leg syndrome (Hening and Caivano 2008), a neurological dysfunction of the dopaminergic system. These findings support the hypothesis of a causal relationship between RA and the dopaminergic system. However, a potential impact of dopaminergic agents in RA patients has been insufficiently investigated so far.

## Dopamine Receptors and Dopaminergic Signaling

Dopamine is a neurotransmitter of the central nervous system controlling movement, emotion, cognition, and neuroendocrine interactions. Dopamine acts on five different dopamine receptors (DR) belonging to the 7-transmembrane, G protein–coupled receptor (GPCR)-family, which are grouped into 2 families: the D1-like dopamine receptors, D1- and D5-DR, which activate adenylate cyclase, and the D2-like dopamine receptors D2-, D3-, and D4-DR, which inhibit adenylate cyclase (Beaulieu and Gainetdinov 2011). Apart from the canonical regulation of cAMP, several studies have shown that DR can also regulate a variety of alternate signaling pathways, such as alternate G protein coupling or non-G protein mechanisms (summarized in (Beaulieu et al. 2015)). A further complexity is the existence of receptor heteromers. It is described that D1-like DR can form heteromers with D2-like DR, and that DR can also form heteromers with several other receptors, such as other GPCRs and with ionotropic receptors (Perreault et al. 2014). The heteromerization confers to the receptor complex a different signaling mechanism compared to the ones activated by the two receptors individually (summarized in (Perreault et al. 2014)). The receptor complex and the intracellular signaling pathway can vary in different organs and cells. This could explain the contradictory results obtained in many studies where single dopamine receptors and specific ligands were investigated. Despite the large complexity added by this new knowledge, future pharmacological strategies could profit from the possibility to target specific receptor heteromers.

## Role of the Dopaminergic Pathway on the Immune Response

Dopamine can modulate the immune system either indirectly, via the modulation of prolactin release, or directly, via binding of dopaminergic receptors on immune cells. In the central nervous system, dopamine can effectively inhibit the release of the peptide hormone prolactin (Borba et al. 2018). Prolactin can bind to its receptor on immune cells and modulates their function (Borba et al. 2018; Buckley 2001; Savino 2017). For instance, prolactin promotes T cell maturation (Carreno et al. 2005) and modulates CD4+ T cell response in a dose-dependent manner (Tomio et al. 2008). Moreover, prolactin can decrease the threshold for B cell activation and increase antibody production, thus promoting autoimmunity (Saha et al. 2009; Peeva and Zouali 2005). In RA, prolactin is increased in the serum and in the synovial fluid, and is responsible for the activation of synovial macrophages (Fojtikova et al. 2010; Abstracts from the European Workshop for Rheumatology Research 2014). Due to its inhibitory effect on prolactin production, one would expect an anti-inflammatory effect of dopamine via inhibition of prolactin. However, treatment with the dopaminergic agonist bromocriptine shows contradictory results (see below), probably due to the fact that prolactin can be also produced in the periphery by immune cells, and this peripheral prolactin synthesis seems to be differently regulated compared to the pituitary gland (Salesi et al. 2013; McMurray 2001).

Besides the indirect effects of dopamine on the immune system via prolactin, dopamine can also directly modulate the immune system, as immune cells express dopaminergic receptors (DR). Experimental evidences have demonstrated that human immune cells express almost all DR (recently summarized in (Arreola et al. 2016)). Among all leukocytes, T cells and monocytes have the lowest DR expression whereas B cells and NK cells have a higher DR expression. Human NK cells express D2-D5DR and lack D1DR (McKenna et al. 2002; Mikulak et al. 2014). Mikulak et al. (Mikulak et al. 2014) reported that dopamine modulates cell function of IL-2-pre-activated NK cells, leading to a dose-dependent reduction of cell proliferation and IFN-α secretion. Human B cells express all DR (McKenna et al. 2002; Ferrari et al. 2004; Meredith et al. 2006). Germinal centre and memory B cells abundantly express D1DR, D3DR and D5DR, and stimulation of dopaminergic receptors results in the differentiation of B cell to plasma cells and a rapid translocation of ICOSL to the cell membrane, thus maximizing T-B cell interaction in the germinal centre (Papa et al. 2017). Of interest, these mechanisms are not conserved between mice and humans (Papa et al. 2017). The required dopamine is released by T follicular helper cells, thus confirming that non-neuronal cells can use dopaminergic pathways independent from the central nervous system (Papa et al. 2017).

The expression of DR in human T cells is very well described (for recent summary, see (Arreola et al. 2016; Levite 2016)). Dopamine usually activates resting human T cells and inhibits activated T cells. However, the effects of dopamine on T cells can be very different and even opposite, depending on the activation state of the cells, the concentration of dopamine and the DR bound by dopamine on the cells (Levite 2016).

Human monocytes show a high expression of D2DR and D3DR, and lower expression of D4DR and D5DR (McKenna et al. 2002). Activation of DR in human monocytes dose-dependently modulates cell proliferation and LPS-mediated activation of NF-kB signaling (Bergquist et al. 2000), and DR activation in human macrophages dose-dependently modulates the secretion of cytokines (Gaskill et al. 2012). The dose-dependent differences of dopamine effects and the discordant results between activated and non-activated cells suggest that dopamine may have different roles in the physiologic and pathologic environment.

Of interest, non-neuronal cells are also able to synthesize dopamine by themselves and to use it for autocrine and paracrine modulation of cell function (Beaulieu and Gainetdinov 2011; Papa et al. 2017; Capellino et al. 2010; Cosentino et al. 2007; Qiu et al. 2004; Jiang et al. 2006; Cosentino et al. 2002; Bergquist et al. 1994; Marino et al. 1999).

In summary, immune cells can be modulated by dopamine because they express DR. The precise effects of the dopaminergic receptors are sometimes controversial. This could be due to the fact that the dopaminergic compounds used in the cited studies have different binding affinities to the DR, as summarized in Table 1, or it could be due to the presence of different DR heteromers with different intracellular pathways compared to single DR, as described above (Perreault et al. 2014). Moreover, DR expression may vary in pathological situations, thus changing the dopaminergic effects on immune cells subpopulation.

**Table 1:** Summary of the pharmacological properties of the cited dopaminergic drugs on human immune cells

Dopaminergic drugs	Cited in ref.	Binding affinity (Ki [nM])	Pharmacological property	Described effects on human immune cells
SCH23390	27	D1-DR (0.2), D5-DR (0.3), 5HT_2C_ (6.3–9.3)	Antagonist (DR) or agonist (5-HT)	NK cells: Reverts the inhibitory effects of dopamine on IFN-γ production. CD4+ T cells: counteracts the dopamine-dependent upregulation of IL-6 and IL-17 (ref: PMID 21307293). PBMC: retards the TPA-induced expression of TH (ref. PMID 15104239). Macrophages: blocks the inhibitory effect of methamphetamine on anti-HIV activity (ref. PMID 23751096). No effects on dopamine-dependent osteoclastogenesis (ref. PMID 23631878)
SKF 38393	27, 30	D1-DR (0.1–1), D5-DR (0.5), D2-DR (150)	Partial agonist	NK cells: strongly inhibits proliferation. Inhibition of IFN-γ expression. Increase in miR-29a expression. Germinal centre B cells: ICOSL upregulation. B cells: no effects on cAMP (ref. PMID 8861180). PBMC: increases the TPA-induced expression of TH (ref. PMID 15104239) Macrophages: no effects on osteoclastogenesis (ref. PMID 23631878).
Quinpirole	27	D2-DR (4.8), D3-DR (24), D4-DR (30)	Agonist	NK cells: no effects on proliferation. Downregulation of D3DR and D4DR expression and cAMP content (ref. PMID 23799052). B cells: no effects on cAMP (ref. PMID 8861180). Macrophages: inhibits osteoclastogenesis (ref. PMID 23631878).
7-Hydroxy-DPAT hydrobromide	27	D3-DR (1), D2-DR (10)	Agonist	NK cells: no effects on proliferation. T cells: strongly increases cell adhesion to fibronectin (ref. PMID 11745370).
PD 168077 maleate	27	D4-DR (8.7)	Agonist	NK cells: no effects on proliferation.
L-741,626	27	D2-DR (2.4)	Antagonist	NK cells: (in combination with U-99194A and L-741,742) Reverts the inhibitory effects of dopamine on IFN-γ production.
U-99194A	27	D3-DR (160), D2-DR (2281)	Antagonist	NK cells: (in combination with L-741,626 and L-741,742) Reverts the inhibitory effects of dopamine on IFN-γ production.
L-741,742	27	D2-DR (2.4), D3-DR (100), D4-DR (220)	Antagonist	NK cells: (in combination with U-99194A and L-741,626) Reverts the inhibitory effects of dopamine on IFN-γ production.
SKF 83566	30	D1-DR (0.56), 5-HT_2_ (11)	Antagonist	Germinal centre B cells: Counteracts the effects of SKF38393 on ICOSL.
Haloperidol	30	D2-DR (1.2), D3-DR (7), D4-DR (2.3), D1-DR (80), D5-DR (100)	Antagonist	NK cells: counteracts the effect of quinpirole on D3DR and D4DR expression and cAMP content (ref. PMID 23799052). Germinal centre B cells: Counteracts the effects of dopamine on ICOSL. Plasma cells: dopamine-dependent differentiation blocked. Macrophages: blocks the dopamine-dependent osteoclastogenesis (ref. PMID 23631878). Inhibits cell adhesion and phagocytosis (ref. PMID 23981042). T cells: IL-4 reduction (ref. PMID 21763349). Counteracts dopamine-dependent cell adhesion to fibronectin (ref. PMID 11745370).
Cabergoline	41, 42	D2-DR (0.6–0.9), D3-DR (0.79), 5HT_2B_ and 5HT_2C_ (1.17), 5HT_2A_ (6.17), 5-HT_1D_ (8.71)	Agonist	To the best of our knowledge, n.d. in human immune cells in vitro.
Bromocriptine	43, 44, 45	D2-DR (0.62–5), α1D-AR (1.12), α1B-AR (1.38)	Agonist	NK cells (in vivo): increases the capacity to recycle after killing (ref. PMID 1400902). B cells: it inhibits cell proliferation and immunoglobulin production in pre-activated B cells (ref. PMID 7688676) PBMC: no effects on TPA-induced TH expression (ref. PMID 15104239) T cells: strongly increases cell adhesion to fibronectin (ref. PMID 11745370).
Pergolide	55	D2-DR (4–50), 5HT_1A_ (1.8–4)	Agonist	T cells: induces cell adhesion (ref. PMID 11745370).

## Dopamine and RA: State of the Art

### Dopaminergic Agents and their Effects in RA: Evidences from the Clinic

Dopaminergic agents were analyzed in the past for the treatment of RA, based on the fact that the stimulation of D2-like DR leads to the inhibition of prolactin, a proinflammatory hormone that is released by the anterior pituitary gland (McMurray 2001) and that is present at high concentration in the serum and synovial fluid of RA patients (Borba et al. 2018; Fojtikova et al. 2010). In these studies, the effect of dopaminergic agonists on the inflammatory process was supposed to be indirect and mediated by prolactin. However, the results of the studies were not congruent (see Table 2).

**Table 2:** Clinical evidences of dopaminergic modulation on RA disease

Reference	Investigated dopaminergic agent	Number of RA patients investigated	Described effects
Mobini M et al. Iran Red Crescent Med J 2011;13:749–50	Cabergoline (D2-like agonist)	patients with active RA (*n* = 10)	Improvement of tender and swollen joint count, patient assessment of pain and patient global assessment of disease activity
Erb N et al., Rheumatology (Oxford). 2001;40:237–9	Cabergoline (D2-like agonist)	one female RA patient	Drastic improvement of the disease parameters
Mader R, Harefuah. 1997;133:527–9, 591	Bromocriptine (D2-like agonist)	patients with refractory RA (*n* = 5)	Some patients profited from the BRC treatment. Results inconclusive due to the small amount of patients.
Figueroa FE et al., Br J Rheumatol. 1997;36:1022–3	Bromocriptine (D2-like agonist)	Female RA patients (*n* = 9)	Overall improvement of disease parameters after treatment.
Dougados M et al., Arthritis Rheum. 1988;31:1333–4	Bromocriptine (D2-like agonist)	RA patients (*n* = 6)	The addition of bromocriptine did not potentiate the effect of CsA therapy. Results inconclusive due to the small amount of patients.
Eijsbouts A et al., J Rheumatol. 1999;26:2284–5	Quinagolide (D2-DR agonist)	RA patients (n = 9)	No beneficial effects of quinagolide on RA

Cabergoline, a D2-like agonist, showed a drastic improvement of the disease parameters in two studies, but in a very limited amount of patients (Mobini et al. 2011; Erb et al. 2001). Bromocriptine, another D2-like agonist, was used in several studies, with contradictory results (McMurray 2001). Figueroa et al. described an improvement of the clinical parameters in RA patients after bromocriptine treatments (Figueroa et al. 1997), whereas Mader described an improvement only in some of the patients (Mader 1997). Dougados et al. hypothesized that reducing the prolactin level via bromocriptine could have a synergistic effect on the immunosuppressive capacity of cyclosporine A (CsA), but they found that in five out of six patients the addition of bromocriptine did not potentiate the anti-inflammatory effect of the CsA therapy, nor reduced the required dosis of CsA (Dougados et al. 1988). A study from Eijsbouts et al. described the treatment of 9 RA patients with quinagolide, another D2-agonist (Eijsbouts et al. 1999), and observed no beneficial effects.

In general, these studies were intended as pilot studies and included a limited number of patients, therefore it is difficult to make any conclusive statement. Moreover, it is difficult to compare studies using different dopaminergic drugs, as they have diverse affinity to DRs and sometimes they can also bind other receptors, thus causing also non-dopaminergic effects, as summarized in Table 1. In general, one can conclude that the modulation of dopamine pathway seems to modulate disease parameters in RA.

Within the last decades, it became clear that DR are also expressed in immune cells and synovial cells in RA, as outlined below. It is therefore plausible that the above-described effects of D2-agonists in RA were also due to a direct interaction of the drugs with immune cells and synovial cells and not solely because of the antagonizing effect on prolactin.

### Involvement of the Dopaminergic System in RA Patients: Experimental Evidences

Besides the clinical evidences, an involvement of dopamine in RA was also described in vitro (see Table 3). For instance, in RA patients a local, high concentration of dopamine was measured in the synovial fluid (Nakano et al. 2011) and it was demonstrated that synovial cells are able to produce and release dopamine (Capellino et al. 2010), thus suggesting that the dopaminergic pathway might represent a non-canonical mechanism in the modulation of local joint inflammation. In a previous study, we could demonstrate that the number of synovial fibroblasts positive for DR was significantly higher in RA compared to osteoarthritis (OA) patients, and the activation of DR via dopamine led to a reduction of IL-6 and IL-8 release from synovial fibroblasts in RA patients not treated with any disease modifying anti-rheumatic drug (DMARD) (Capellino et al. 2014). The treatment of mixed synovial cells with reserpine, which induces a rapid release of the stored dopamine (together with noradrenaline) from the cells, led to a strong inhibition of TNF release in RA patients (Capellino et al. 2010). D2-like DR were described also on B cells in the synovium of RA patients and in mast cells in the synovial fluid. The amount of D2DR^+^ B cells in the synovial tissue was higher in RA compared to OA (Wei et al. 2016), whereas the number of D3DR^+^ mast cells was negatively correlated to disease severity in RA (Xue et al. 2018). Unfortunately, the effect of D2-like DR activation in these cells was not investigated. In the blood, the amount of D2DR^+^ B cells positively correlates with TNF levels in RA, thus suggesting an involvement of D2DR^+^ B cells also on systemic inflammation (Wei et al. 2016). Taken together, these results suggest that the dopaminergic pathway is involved in RA and is able to modulate the local as well as the systemic inflammation. However, given the current data it is difficult to assign a definite proinflammatory or an anti-inflammatory role for dopamine in RA. More detailed analysis of dopamine-modulated pathways in immune cells and synovial cells during arthritis are still required. Moreover, it was demonstrated that G protein coupled receptors such as DR can switch from Gαs to Gαi signaling during chronic inflammation in RA synovium (Jenei-Lanzl et al. 2015). Therefore, one can assume that the effects of DR activation on arthritis could vary during the disease. Thus, disease duration and disease activity should be taken under consideration to better interpret the results of dopaminergic effects in arthritis.

**Table 3:** Effects of dopaminergic modulation on RA patients: experimental evidences

Reference	Investigated dopaminergic pathway	Target tissue/cells	Described effects
Xue L et al., Clin Rheumatol. 2018 Jun 22	D3DR	Mast cells from the synovial fluid	Negative correlation between D3R-positive mast cell number in the synovial fluid and disease severity (DAS28 score) of RA patients
Wei L et al., BMC Musculoskelet Disord. 2016;17:352	D2DR	Peripheral and synovial B cells	More DR2(+)CD19(+) B cells in synovial tissues from RA patients than in those from osteoarthritis (OA) patients. The frequency of peripheral B cells expressing DR2 positively correlated with plasma TNF-α level
Capellino S et al., Arthritis Rheumatol. 2014;66:2685–93	all DRs	Synovial fibroblasts	DR are strongly expressed and dopamine synthesized in RA synovial fibroblasts. Exogenous dopamine strongly inhibited the production of IL-8 in RA
Nakano K et al., J Immunol. 2011;186:3745–52	Dopamine synthesis, Dopamine, D1-like DR	Peripheral T cells, Synovial tissue	Levels of dopamine are higher in RA synovial fluid compared to OA. Dopamine leads to increased secretion of proinflammatory cytokines from human peripheral T cells
Capellino S et al., Ann Rheum Dis 2010;69:1853–60	Dopamine synthesis and release	Synovial cells	Synovial cell of RA patients synthesize and release dopamine. Treatment of synovial cells with reserpine led to a strong inhibition of TNF release.

### Dopaminergic Agents in Animal Models of Arthritis

A potential direct role of dopamine was investigated in several in vivo and in vitro studies in animal models of arthritis (Table 4). D2-like receptor activation led to reduced cartilage destruction and synovial hyperplasia in SCID mice engrafted with human synovium (Nakano et al. 2011) as well as in the collagen-induced arthritis (CIA) mouse model (Lu et al. 2015). Also, Drd2 (−/−) mice manifested a more severe CIA compared to wild-type mice (Lu et al. 2015). In vitro*,* the stimulation of D2-like DR had anti-inflammatory effects on lymphocytes from CIA mice (Lu et al. 2015). Besides the effects on inflammation, D2DR seem to be involved also in nociception in mice (Robledo-Gonzalez et al. 2017). In rats, the role of D2-like DR is controversial. The blockade of D2-like DR in the CIA model reduced the amount of proinflammatory biomarkers, thus suggesting a proinflammatory role of D2-like DR (Fahmy Wahba et al. 2015). In contrast, the treatment with pergolide, a DR agonist with higher affinity to D2-like than to D1-like DR, led to anti-inflammatory effects in the carrageenan-induced arthritis model (Bendele et al. 1991).

**Table 4:** Dopaminergic modulation of arthritis in the animal models

Reference	Dopaminergic agent	Tissue/cells	Animal model	Described effects
In vivo*:*				
Zhu H et al., PLoS One. 2017;12(9):e0183484	Carbidopa (inhibits peripheral metabolism of levodopa)	T cells	Collagen-induced arthritis (CIA) in mice	Carbidopa strongly inhibited T cell activation in vitro and in vivo and mitigated collagen-induced arthritis
Robledo-González LE et al., J Pain Res. 2017;10:1777–86	Mazindol (inhibits DA reuptake)	Bone	CFA-induced knee arthritis in mice	Mazindol via D2-like receptors has an antinociceptive role in mice with CFA-induced knee arthritis without modifying the bone density
Lu JH et al. Biomed Res Int. 2015;2015:496759	D2DR/Quinpirole (agonist)	Joints and Serum	Collagen-induced arthritis (CIA) in mice	Quinpirole intraperitoneal administration reduced both clinical arthritis score and serum anti-CII IgG level in CIA mice. Drd2 (−/−) CIA mice manifested more severe limb inflammation and higher serum anti-CII IgG level and further upregulated anti-inflammatory cytokine expression and downregulated proinflammatory cytokine expression than wild-type CIA mice
Fahmy Wahba MG et al. Eur J Pharmacol 2015;765:307–15	D2DR/ Haloperidol (antagonist)	Serum	Collagen-induced arthritis (CIA) in female rats	Rheumatoid factor, matrix metalloprotinease-3, serum immunoglobulin G, antinuclear antibody as well as some immunological biomarkers were back to normal in haloperidol-treated CIA rats.
Nakano K et al., J Immunol 2011;186:3745–52	Haloperidol (D2-like antagonist) and SCH23390 (D1-like antagonist)	Synovium and cartilage	SCID mice engrafted with human RA synovium	Less cartilage destruction and synovial hyperplasia in SCH23390-treated animal. Opposite effects in haloperidol-treated animals.
Nakashioya H et al., Mod Rheumatol. 2011;21:260–6	SCH23390 (D1-like antagonist)	Joints and Serum	Collagen-induced arthritis (CIA) in DBA/1 mice	Lower CIA severity in treated mice, but no effects on the serum level of antibodies to collagen type-II
Bendele Am et al., J Pharmacol Exp Ther. 1991;259:169–75	Pergolide (DR agonist, D2 > D1)	Joints	Carrageenan paw edema assay in rats	Pergolide had anti-inflammatory effects, probably due to the activation of central DRs.
In vitro*:*				
Lu JH et al. Biomed Res Int. 2015;2015:496759	D2DR/Quinpirole (agonist)	Lymphocytes	Collagen-induced arthritis (CIA) in mice	Quinpirole downregulated expression of proinflammatory Th17-related cytokines but further upregulated expression of anti-inflammatory Treg-related cytokines
Nakashioya H et al., Mod Rheumatol. 2011;21:260–6	SCH23390 (D1-like antagonist)	Bone marrow-derived macrophages	Collagen-induced arthritis (CIA) in DBA/1 mice	Osteoclast differentiation is reduced after SCH23390 treatments. No alteration of inflammatory cytokine expression.

The in vivo D1-like DR blockade showed proinflammatory effects on arthritis in mice (Nakano et al. 2011; Nakashioya et al. 2011). In vitro, blockade of D1-like DR led to reduced osteoclast differentiation in CIA mice, but no alteration of inflammatory cytokines was observed (Nakashioya et al. 2011).

Due to the effects of dopaminergic drugs on the immune system, drugs used for the treatment of Parkinson can also alter arthritis onset and progression, as recently described by Zhu et al. (Zhu et al. 2017). In this study, Zhu et al. investigated the effect of carbidopa, a drug able to block the conversion of levodopa to dopamine in the periphery and therefore used in combination with levodopa in Parkinson’s patients. Their results showed that the intake of carbidopa decreased joint inflammation and arthritis score in CIA mice (Zhu et al. 2017).

Taken together, knowledge from animal studies strongly corroborate the hypothesis that dopaminergic drugs could be beneficial to treat arthritis. However, some crucial points remain to be clarified. For example, it has not been fully elucidated if the dopaminergic drugs have any neurological side effects on the animals if administered systemically. Moreover, it is difficult to compare results from different animal models of arthritis and using different drugs acting on the different classes of DR. More detailed studies will be required to better determine the mechanisms of action of the dopaminergic pathway in arthritis in vivo, but the current results are already very promising and suggest a new therapeutic option for arthritis.

## Future Perspectives for Dopaminergic Drugs in Rheumatoid Arthritis

Current results suggest a direct involvement of the dopaminergic pathway on the immune response in rheumatoid arthritis. Therefore, the use of dopaminergic drugs could represent a promising alternative therapeutic strategy in arthritis patients. However, there are still several issues that need clarification prior assigning the role of the “bad guy” or the role of the “good one” to specific DR in RA. For instance, it is necessary to determine the signaling pathway involved in DR activation in immune cells and in synovial cells during arthritis, and investigate if specific DR always act proinflammatory (or anti-inflammatory) or if they can switch their intracellular signaling due to the chronic inflammation or due to the formation of receptor heteromers (Perreault et al. 2014; Jenei-Lanzl et al. 2015). If this were the case, it would be necessary to determine how the disease stage correlates with the effect of specific DR. Moreover, due to possible side effects on the central nervous system, a cell- (or tissue-) targeted modulation of DR would be preferable, but its efficacy still needs to be investigated. Another crucial point is the possible interactions of DMARD and dopaminergic drugs. Indeed, in patients affected by multiple sclerosis it is described that the treatment with IFN-beta leads to the loss of function of dopamine on T cells (Cosentino et al. 2012), and it is plausible that such alterations of the dopaminergic pathways could also occur in RA patients treated with DMARD. Therefore, a possible influence of the therapy with DMARD on dopamine-related immune response needs to be addressed in the future.

In summary, the current knowledge is encouraging and supports fascinating future possibilities for the use of dopaminergic drugs for treating arthritis, after more intensive research on this topic. Nevertheless, clinicians should already now be aware of a probable influence of dopamine on the immune response in arthritis when treating RA patients with dopaminergic drugs due to comorbidities, and possible unexpected effects on the immune system and on disease progression should be carefully monitored.
